# Pachydermoperiostosis or primary hypertrophic osteoarthropathy: A rare clinicoradiologic case

**DOI:** 10.4103/0971-3026.50829

**Published:** 2009-05

**Authors:** Rajul Rastogi, GN Suma, Ravi Prakash, Umesh Chandra Rastogi, Sumeet Bhargava, Vaibhav Rastogi

**Affiliations:** Yash Diagnostic Center, Yash Hospital and Research Center, Civil Lines, Kanth Road, Moradabad, Uttar Pradesh-244 001, India; 1 Kothiwal Dental College and Research Center, Kanth Road, Moradabad, Uttar Pradesh-244 001, India

**Keywords:** Hypertrophic osteoarthropathy, pachydermoperiostosis

## Abstract

Pachydermoperiostosis (PDP) or primary hypertrophic osteoarthropathy is a rare syndrome with diverse radiological and clinical features. Though the diagnosis can be made on the basis of the classic clinical and radiological features, it is often missed due to variable presentations. A case of PDP that presented with dental complaints and had almost all the clinical and radiological features described in literature is reported. We also discuss the differential diagnosis.

Pachydermoperiostosis (PDP) is a rare osteo-arthro-dermopathic syndrome with familial and idiopathic forms differentiating it from secondary / pulmonary hypertrophic osteoarthropathy.[1] Coexistence of the features of periostosis and cutaneous thickening along with the absence of any disease involving any other system of the body suggests PDP. According to one study, it has an estimated prevalence of 0.16%.[2] It usually manifests in adolescence, occurring almost exclusively in males, with a M: F ratio of 7:1. It is associated with significant morbidity with advancing age.[3,4] Rarely, as happened in our case, it may remain undiagnosed and progress until there are significant facial, joint, and digital deformities that finally make the patient seek medical attention.

## Case Report

**Figure 1 (A,B):**
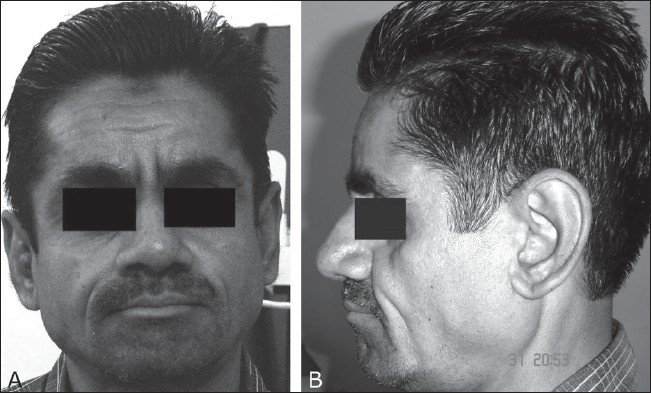
Clinical photograph of the face from the front (A) and the left side (B) shows prominent skin folds on the forehead and cheek

A 40-year-old male presented to the dental department with swelling of the gums. Clinically, the patient had periodontal disease; anemia; clubbing, and acromegaly; deformities of the hands and feet; swelling without acute inflammatory signs at the wrist, ankle, and knee joints; and thickening and folding of the facial skin [Figures 1]. Lingual enlargement was not noted. The patient had hyperhidrosis and complained of a feeling of heat in the palms and soles. He denied any history of trauma. A family history of similar disease was negative. To exclude any systemic cause for the periodontal disease, the patient was referred to the radiology department for radiographs of both hands and feet and both forearms and legs. Laboratory examination, including thyroid profile, growth hormone assay, tests for syphilis, and smears of skin for AFB, were unremarkable. The ESR was moderately elevated, the hemoglobin was reduced, and cytology of synovial fluid from one of the knee joints showed features of chronic noninfectious inflammation.

**Figure 2 (A,B):**
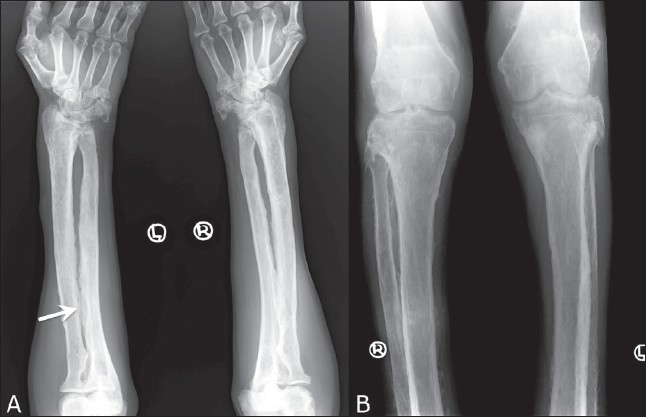
Anteroposterior radiographs of both forearms (A) and upper legs (B) show symmetric, shaggy periosteal proliferation extending up to the epiphyses, with expansion of the radius and ulna at the wrist and of the tibia and fibula at the knee, along with calcification of the interosseous membranes (arrow)

Radiographs of both forearms and legs revealed symmetric, exuberant, shaggy subperiosteal bone formation, along with ossification of the interosseous membranes; this was more prominent in the leg bones [Figure 2]. Additionally, there was expansion of the distal ends of the radius and ulna and the proximal ends of the tibia and fibula, which was associated with a reduction in the radiocarpal and femorotibial joint spaces.

**Figure 3:**
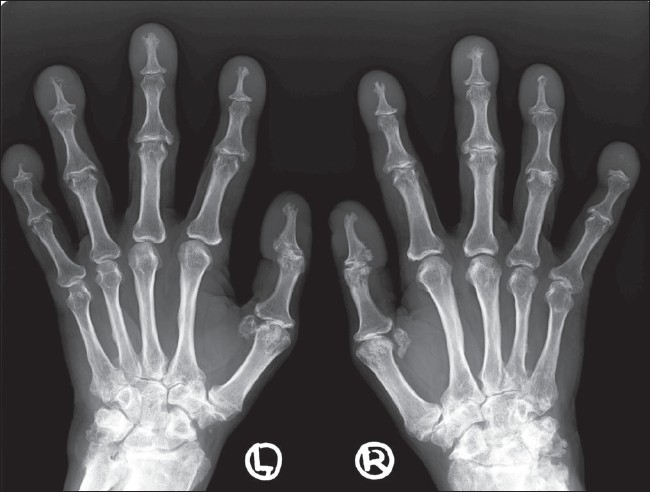
Anteroposterior radiograph of both hands shows enlargement of the distal fingers with acroosteolysis, along with modeling deformities of the sesamoid bones, reduced joint spaces at the carpus and the proximal and distal interphalangeal joints, periarticular osteopenia, and joint deformities

Radiographs of both hands and feet, including the wrists and ankle joints, revealed widening of the epiphysis and exuberant subperiosteal bone formation, especially at the distal ends of the forearm and leg bones and the metacarpal and metatarsal bones. There was associated increase in osseous density of the carpal and tarsal bones, and the metacarpal, metatarsal, and phalangeal bones, along with variable reduction in the joint spaces of the proximal interphalangeal (PIP) and distal interphalangeal (DIP) joints; there was also periarticular osteopenia and resorption of the distal phalanges, associated with soft tissue swelling of the distal fingers and toes and contractures of the toes. There was evidence of periarticular erosions at the PIP joints. Additionally, there was enlargement and modeling deformities of the sesamoid bones in both hands and feet with deformities at multiple joints; these changes were more severe in the feet [Figure 3]. Besides, there was evidence of collapse of the calcaneus.

**Figure 4:**
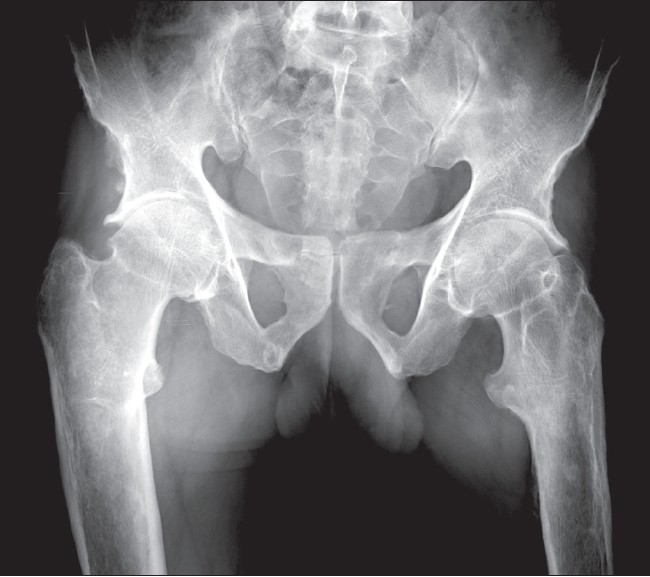
Anteroposterior radiograph of the pelvis (including both hips) shows symmetric and shaggy periosteal proliferation in the lower part of the iliac bones. There is widening of the femoral metaphyses with reduction of hip joint space on both sides, without any abnormality of the articular surface

Radiograph of the pelvis, including both hip joints, revealed exuberant new bone formation along the iliac bones. Cortical thickening with widening of the femoral shafts was noted bilaterally. There was reduction in the hip joint spaces bilaterally but the articular surfaces were smooth [Figure 4].

**Figure 5:**
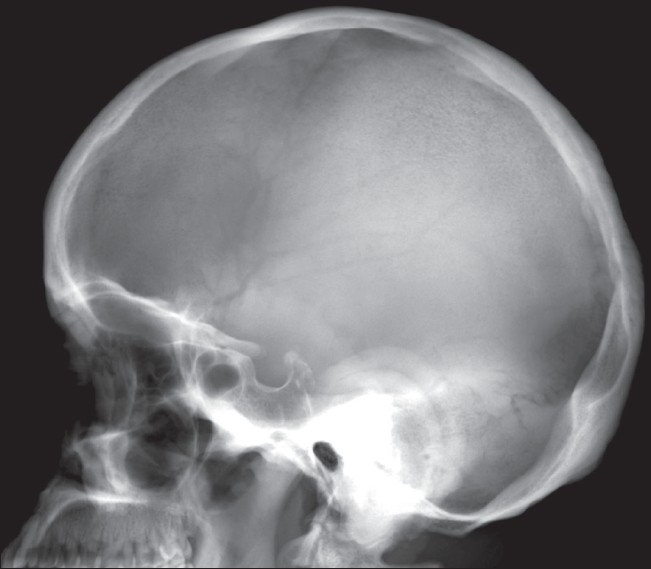
Lateral radiograph of the skull shows hyperostosis of the calvaria and skull base bones with a normal sella and sinuses

A lateral radiograph of the skull revealed hyperostosis of the skull bones; however, the sella turcica appeared normal [Figure 5]. A radiograph of the chest was unremarkable, while that of the spine revealed spondylotic changes with loss of lumbar lordosis.

**Figure 6:**
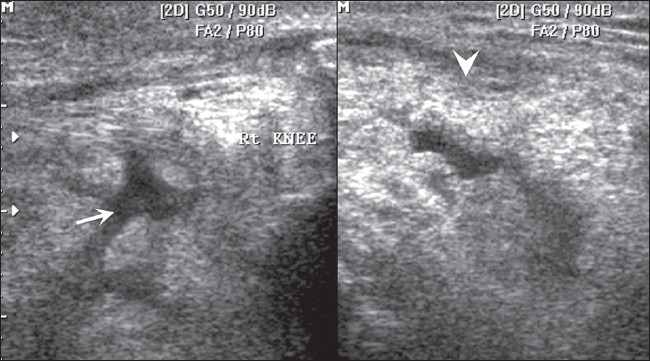
USG of the right knee joint at the level of the suprapatellar bursa shows fluid (arrow) and a thickened synovium (arrowhead)

USG examination of the knee and ankle joints revealed mild effusion with thickened synovium bilaterally [Figure 6].

Based on the clinical and radiological findings, a diagnosis of the classic or complete form of PDP or idiopathic hypertrophic osteoarthropathy was suggested. Histopathological examination of the skin demonstrated an increase in dermal collagen, hyperkeratosis, and acanthosis, with no evidence of any acid-fast bacilli.

## Discussion

Hypertrophic osteoarthropathy is a triad comprising clubbing of digits, subperiosteal bone formation, and mono- or polyarthritis.[5] PDP is a rare form of hypertrophic osteoarthropathy with no known cause and hence is often called idiopathic or primary hypertrophic osteoarthropathy to distinguish it from secondary or pulmonary hypertrophic osteoarthropathy. PDP accounts for 3–5% of cases of hypertrophic arthropathy.[6,7] The secondary form results from cardiopulmonary diseases (e.g., bronchiectasis, cystic fibrosis, congenital heart diseases, and tuberculosis); hepatic diseases (e.g., portal and biliary cirrhosis); gastrointestinal diseases (e.g., inflammatory bowel disease and polyposis); and certain malignancies (e.g., Hodgkin's disease, carcinoma nasopharynx, and chronic myeloid leukemia).[1,7–9]

In up to one-third of the patients, PDP occurs as a hereditary disease with autosomal dominance of variable penetrance. There may be involvement of the skin, bones, and joints.[4] Some case reports suggest that it may be an X-linked disease.[10] The condition is associated with thickening of the facial skin and scalp (pachyderma) and periarticular and subperiosteal periostosis or bone formation, with consequent enlargement of the hands and feet, joint deformities, and neurological disturbances. Associated clinical features that have been described include clubbing of digits, with or without paronychial thickening; arthralgias; seborrheic dermatitis; mechanical ptosis of thickened eyelids; periodontal disease; and palmoplantar hyperhidrosis.[3] Touraine *et al*. described three forms of PDP, viz, *classic or complete form*, with skin and skeletal changes; *incomplete form*, with skeletal changes but no dermal findings; and *forme fruste* with dermal changes but no skeletal findings.[8]

Facial involvement occurs in the form of thickening of the facial skin and scalp, with prominent folds on the forehead and cheek. A leonine facies is usually a late feature. Sometimes, the scalp takes on an undulating appearance and shows prominent grooves, the appearance being referred to as *cutis verticis gyrata* because of its resemblance to the sulci and gyri of brain. There can also be a so-called *bull-dog appearance*, a feature that was not seen in our case. Cutis verticis gyrata can also been seen in a variety of other conditions, including neurofibromatosis, diabetes mellitus, myxedema, cretinism, amyloidosis, acromegaly, etc., as well as in a variety of other syndromes including Turner's syndrome, Noonan's syndrome, tuberous sclerosis, etc., and hence it is not pathognomonic for PDP.[11]

Skeletal findings include symmetric, shaggy subperiosteal bone formation in the long bones, especially of the forearm and leg bones, but also of the metacarpals, metatarsals, and phalanges. Involvement of the epiphyseal region distinguishes it from the secondary form, in which the epiphyses are usually spared.[7] There is widening of the ends of bones, especially at the wrist and knee joints. Widening is due to increased bone formation, which is concomitant with histological evidence of increased collagen formation and increased urinary excretion of hydroxyproline.[12] Factors that stimulate both osteoblasts and osteoclasts have been implicated, thus explaining both the periostosis as well as the osteoporosis / osteolysis seen in this condition.[13] A prominent feature is enlargement of the distal part of the digits with resorption of the distal phalanges or acroosteolysis and calcification of ligaments and interosseous membranes. In later stages, cortical thickening with narrowing of the medullary cavity may be seen. Enlargement of sinuses may be seen uncommonly. Bone scintigraphy may reveal increased tracer uptake by the cortex in the diaphyseal and metaphyseal regions.[1,3] Hyperostosis of the calvaria and skull base bones, as seen in our case, is common.[7]

Joints affected in PDP show swelling due to joint effusion, with evidence of chronic nonsuppurative inflammation. There is reduction in joint spaces, with relative preservation of articular surfaces. Late-onset deformities, including contractures, may occur, especially in the digits.[1] Periarticular erosions, as seen at the PIP joints in our case, are very rare.[14]

Spinal manifestations are unusual but have been described. These include narrowing of the intervertebral disk spaces and foramina, dense striations in the vertebral bodies arranged in a horizontal or vertical fashion, and ligamentous ossification and laxity with secondary spondylolisthesis.[1,7,8] Spinal findings were not a prominent feature in our case.

Differential diagnoses include variants of PDP, secondary hypertrophic osteoarthropathy, thyroid acropachy, acromegaly, van Buchem's disease (in which there is absence of clubbing and skin changes), diaphyseal dysplasia (endosteal and periosteal proliferation), and syphilitic periostosis.

Variants of PDP include *Rosenfeld-Kloepfer syndrome* (characterized by enlargement of the jaws, especially mandible, and of the hands and feet, nose, lips, tongue, and forehead, along with cutis vertices gyrata and corneal leukoma); *Currarino idiopathic osteoarthropathy* (an incomplete form of PDP seen in children and adolescents and characterized by the presence of eczema and sutural diastases); and a *localized form* with only the radiographic features of PDP in the lower extremities.[3]

Treatment is limited to NSAID, steroid, or colchicine therapy to alleviate arthralgias and retinoids for the dermal changes. Surgical treatment is limited to plastic surgery for cosmetic indications or correction of associated deformities.[1,3,13]

To summarize, the classic or complete form of PDP is rare but presents with the distinct clinicoradiologic features of clubbing and enlargement of digits, thick and coarse facial skin, subperiosteal bone formation in the long bones, and joint abnormalities, including effusion and deformities. Early diagnosis helps in reducing the morbidity and in prognostication.

## References

[1] Kowalewski M, Urban M, Górska A (1997). Familial occurrence of primary hypertrophic osteoarthropathy: A case report. Med Sci Monit.

[2] Jajic I, Jajic Z (1992). Prevalence of primary hypertrophic osteoarthropathy in selected population. Clin Ex Rheum.

[3] Goyal S, Schwartz RA, Richards GM, Goyal R Pachydermoperiostosis.

[4] Sahasrabhojaney VS, Hinge AV, Ghodeswar SS, Machnurkar AS, Daware AM, Nagarik AP (2005). Touraine-Solente-Gole' syndrome. J Indian Acad Community Med.

[5] Carcassi U (1992). History of hypertrophic osteoarthropathy (HOA). Clin Exp Rheumatol.

[6] Eckardt A, Kreitner KF (1995). Primary hypertrophic osteoarthropaty. Z Orthop Ihre Grenzgeb.

[7] Resnick D, Resnick D, Kransdorf MJ (2005). Enostosis, Hyperostosis, and periostitis. Bone and Joint imaging.

[8] Bhaskaranand K, Shetty RR, Bhat AK (2001). Pachydermoperiostosis: Three case reports. J Orthop Surg (Hong Kong).

[9] Gilliland BC, Kasper DL, Braunwald E, Fauci AS, Hauser SL, Longo DL, Jameson JL (2005). Fibromyalgia, arthritis associated with systemic disease, and other arthritis. Harrison's Principle of Internal Medicine.

[10] Sayli U, Yetkin H, Atik OS, Uluoglu O, Bülükbasi S (1993). Pachydermoperiostosis: A case report. J Foot Ankle Surg.

[11] Skibinska MD, Janniger CK (2007). Cutis Verticis Gyrata.

[12] Cooper RG, Freemoni AJ, Relev M, Holt L, Anderson DC, Jayson MI (1992). Bone abnormalities and severe arthritis in pachydermoperiostosis. Ann Rheum Dis.

[13] Auger M, Stavrianeas N Pachydermoperiostosis. Orphanet Encylcopedia.

[14] Schumacher HR (1992). Hypertrophic osteoarthropathy: rheumatologic manifestations. Clin Exp Rheumatol.

